# Volta Phase Plate Cryo-EM Structure of the Human Heterodimeric Amino Acid Transporter 4F2hc-LAT2

**DOI:** 10.3390/ijms20040931

**Published:** 2019-02-21

**Authors:** Jean-Marc Jeckelmann, Dimitrios Fotiadis

**Affiliations:** Institute of Biochemistry and Molecular Medicine, and Swiss National Centre of Competence in Research (NCCR) TransCure, University of Bern, CH-3012 Bern, Switzerland; jean-marc.jeckelmann@ibmm.unibe.ch

**Keywords:** 4F2hc, cryo-EM, direct electron detector, heteromeric amino acid transporter, LAT2, L-type amino acid transporter, SLC3, SLC7, Volta phase plate

## Abstract

Heteromeric amino acid transporters (HATs) are protein complexes that catalyze the transport of amino acids across plasma membranes. HATs are composed of two subunits, a heavy and a light subunit, which belong to the solute carrier (SLC) families SLC3 and SLC7. The two subunits are linked by a conserved disulfide bridge. Several human diseases are associated with loss of function or overexpression of specific HATs making them drug targets. The human HAT 4F2hc-LAT2 (SLC3A2-SLC7A8) is specific for the transport of large neutral L-amino acids and specific amino acid-related compounds. Human 4F2hc-LAT2 can be functionally overexpressed in the methylotrophic yeast *Pichia pastoris* and pure recombinant protein purified. Here we present the first cryo-electron microscopy (cryo-EM) 3D-map of a HAT, i.e., of the human 4F2hc-LAT2 complex. The structure could be determined at ~13 Å resolution using direct electron detector and Volta phase plate technologies. The 3D-map displays two prominent densities of different sizes. The available X-ray structure of the 4F2hc ectodomain fitted nicely into the smaller density revealing the relative position of 4F2hc with respect to LAT2 and the membrane plane.

## 1. Introduction

Amino acids are fundamental nutrients and are involved in numerous cellular processes ranging from energy production to signaling and protein synthesis. Transport of amino acids and amino acid derivatives across the plasma membrane is achieved by amino acid transporters belonging to different solute carrier (SLC) families (available online: http://slc.bioparadigms.org). From these families, the SLC7 family of amino acid transporters is composed of 15 members that belong to the large amino acids, polyamines, and organocations (APC) superfamily of transporters [1]. SLC7 family members are subdivided in two groups, the cationic amino acid transporters (CATs; SLC7A1-4 and SLC7A14) and the L-type amino acid transporters (LATs; SLC7A5-13 and SLC7A15). LATs are glycoprotein-associated amino acid transporters, i.e., the catalytic (light) subunits of these heteromeric amino acid transporters (HATs) are associated with the heavy subunits 4F2hc (SLC3A2) or rBAT (SLC3A1) from the SLC3 family [1]. A conserved disulfide bridge covalently links the two HAT subunits [1,2,3]. The amino acid transporter LAT2 (SLC7A8) preferably transports large neutral L-amino acids and functions as obligatory exchanger [4]. It is associated with the heavy chain subunit 4F2hc (CD98), which is a type II membrane *N*-glycoprotein. 4F2hc comprises a small N-terminal cytoplasmic domain, followed by a single transmembrane domain (TMD) and a large C-terminal ectodomain (ED) [1,5], whose structure was solved [6]. The heavy subunit is responsible for stabilization of the 4F2hc-LAT2 heterodimeric complex and its correct trafficking to the plasma membrane [7,8]. The light subunit LAT2 is highly hydrophobic, not glycosylated, and responsible for substrate transport. It is predicted to contain twelve TMDs with internal N- and C-termini [1,4]. Human nuisances and severe diseases such as age-related hearing loss, renal aminoaciduria, and cancer are associated with a loss of transporter function or overexpression of LAT2 [1,9,10,11].

A few years ago, the heterologous expression of human light and heavy subunits using the methylotrophic yeast *Pichia pastoris* as expression system was reported [12]. The most successfully overexpressing subunits turned out to be 4F2hc and LAT2. Co-transformation and -expression of these two subunits yielded functional complexes of 4F2hc-LAT2 connected by the conserved disulfide bridge [12]. For structural studies, the human 4F2hc-LAT2 heterodimeric complex was overexpressed containing N-terminal affinity tags, i.e., His-tagged 4F2hc and Strep-tagged LAT2, and purified from detergent-solubilized membranes [7,13]. The transport activity of recombinant 4F2hc-LAT2 was confirmed by l-leucine uptake experiments using either Pichia cells or proteoliposomes [7,12]. 3D-maps at about 20 Å resolution of the 4F2hc-LAT2 complex were obtained using single particle analysis and negative-stain electron microscopy [7,13]. These maps provided first insights into the supramolecular organization of human 4F2hc-LAT2 at low resolution.

In recent years, the field of electron microscopy (EM) has undergone a revolution in terms of resolution. The main factors for this are the introduction of improved hardware, e.g., direct electron detectors, and software for image processing and automated data acquisition [14,15,16]. Furthermore, structure solution of difficult protein samples as small, heterogeneous, and flexible proteins is facilitated by the introduction of the Volta phase plate (VPP) device into electron microscopes [17]. This technology increases the contrast of electron micrographs and thus supports the structure solution of relatively small proteins [18]. Here we present the first cryo-EM 3D-map of a HAT, i.e., of the human 4F2hc-LAT2 complex. This structure at ~13 Å resolution was successfully obtained using the direct electron detector and VPP technologies. The available X-ray structure of 4F2hc-ED was fitted into the obtained density of human 4F2hc-LAT2 and revealed the relative positions of the heavy subunit with respect to the light subunit and the membrane plane.

## 2. Results and Discussion

**Figure 1:**
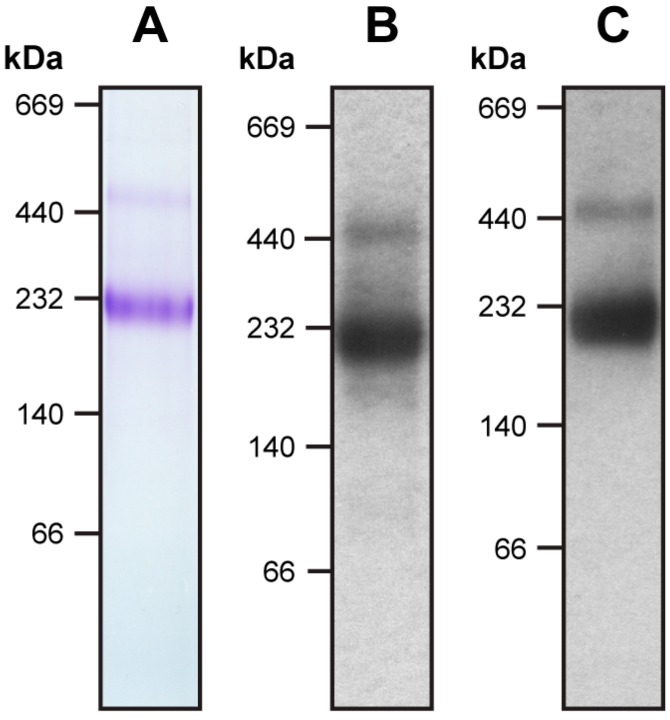
Blue native-PAGE and Western blot analysis of purified human 4F2hc-LAT2 heterodimeric complex. Panel (**A**) displays a Coomassie Brilliant Blue stained 4-16% gradient blue-native gel of purified 4F2hc-LAT2. Western blot analyses using anti-His (**B**) and anti-Strep antibodies (**C**) indicate purity and correct assembly of the complex. The heterodimeric complex migrates to about 230 kDa. The faint band above the 440 kDa marker is in good agreement with a dimer of the heterodimeric complex, possibly due to unspecific aggregation. Note that neither monomeric 4F2hc nor monomeric LAT2 are present in the sample. Five micrograms of purified protein was loaded per lane.

The recombinant 4F2hc-LAT2 complex was overexpressed using the methylotropic yeast *P. pastoris* and purified using Ni-nitrilotriacetic acid (NTA) affinity chromatography from lauryl maltose neopentyl glycol (LMNG)/cholesteryl hemisuccinate (CHS) solubilized membranes. The purified protein complex was pure and correctly assembled as reflected by a prominent band in the Coomassie Brilliant Blue stained blue native (BN)-polyacrylamid gel (Figure 1A). Correct assembly of the complex was further confirmed using Western blot analysis, which revealed the presence of both proteins, 4F2hc and LAT2, in this major band (Figure 1B,C). According to BN-polyacrylamide gel electrophoresis (BN-PAGE), the molecular weight (MW) of the detergent-solubilized, purified complex was estimated at about 230 kDa, whereas the calculated MW of the complex based on the primary amino acid sequences of 4F2hc and LAT2 is about 120 kDa. Therefore, the LMNG/CHS micelle of the protein complex and co-purified lipids accounts for almost halve of the total MW. On the basis of the primary amino acid sequences, an approximate two-fold increase in MW of membrane transport proteins containing a comparable amount of TMDs as 4F2hc-LAT2, analyzed using BN-PAGE, were reported, supporting our result [19,20]. Inspection of the gel revealed a faint secondary band migrating at higher MW, i.e., at about 450 kDa. Western blot analysis clearly showed that both proteins, 4F2hc and LAT2, are present in this band (Figure 1B,C). This hints at the formation of dimers of 4F2hc-LAT2 heterodimeric complexes, possibly due to unspecific aggregation as previously reported [7,13]. Detergent-solubilized 4F2hc and LAT2 originating from disrupted heteromeric complexes are expected to be smaller in size than its complex and would therefore migrate to lower MWs than 230 kDa (for SDS-PAGE analysis of 4F2hc-LAT2 under reducing and non-reducing conditions see [7,12,13]). Since no protein bands below 230 kDa were detected using BN-PAGE and Western blot analysis (Figure 1), the use of LMNG/CHS detergent mixture for purification turned out to be beneficial for 4F2hc-LAT2 heterodimer stability.

**Figure 2:**
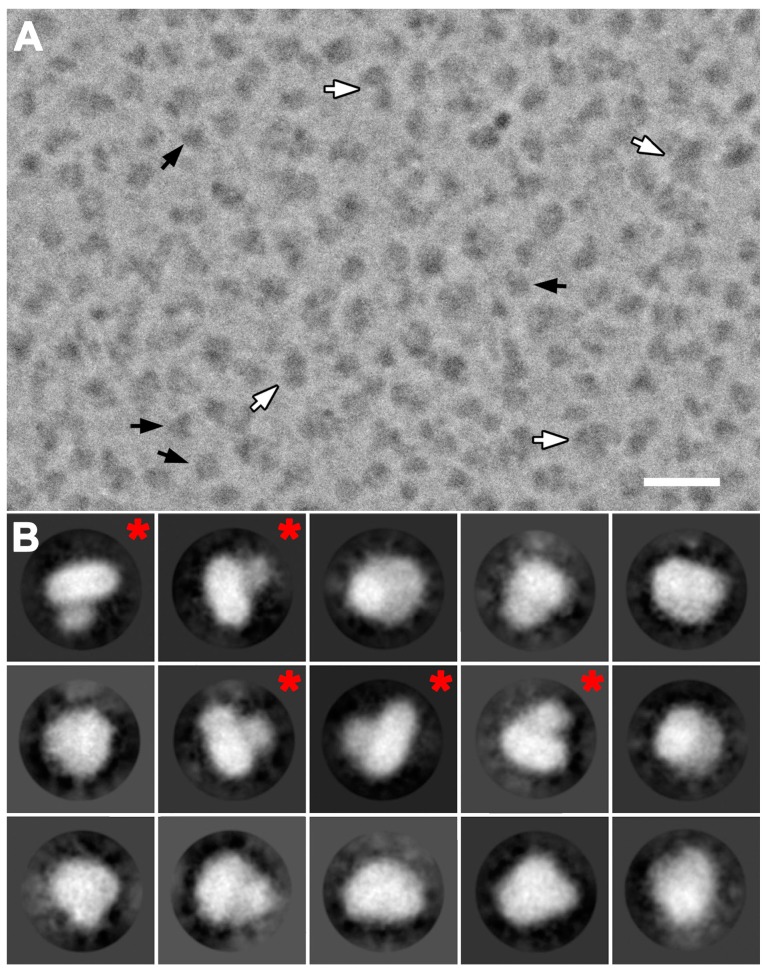
Cryo-electron microscopy (cryo-EM) of purified 4F2hc-LAT2 and gallery of 2D-class averages. (**A**) Displayed is a representative cryo-EM image collected using the Volta phase plate (VPP). Two sets of particles with different lengths are discerned. Smaller particles are in a size range of 100–120 Å (black arrows) and significantly more abundant than larger particles (size range of 180–200 Å, white arrows). (**B**) A gallery of 15 representative 2D-class averages is displayed and ordered according to decreasing number of particles assigned to each class. 2D-class averages labeled with a red asterisk reflect projections as viewed from the membrane plane indicating two differently sized densities. Protein density is black and white in panels (**A**) and (**B**), respectively. The scale bar in (**A**) is 300 Å and the frame size in (**B**) 227 Å.

**Figure 3:**
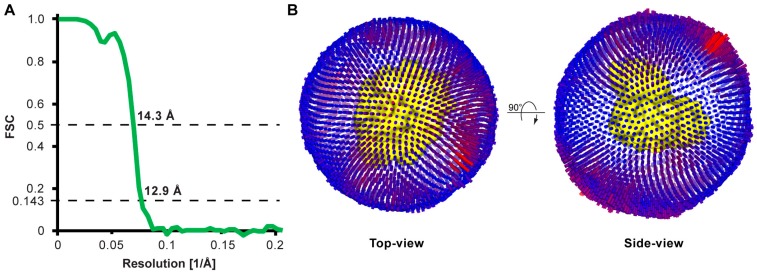
Resolution estimation and angular particle distribution. (**A**) The Fourier shell correlation (FSC) plot for the calculated 4F2hc-LAT2 3D-map indicated resolutions of 14.3 Å and 12.9 Å at cut-offs of 0.5 and 0.143 (gold standard), respectively. (**B**) Angular distribution plots of particles included in the final 3D reconstruction viewed from the top and the side. The number of particles with respective orientations are represented by length and colored cylinders, ranging from blue to red. In the center, the cryo-EM 3D-map of 4F2hc-LAT2 is represented as yellow colored surface.

Cryo-EM images of purified 4F2hc-LAT2 particles were initially recorded with a 200 kV FEI Tecnai F20 electron microscope equipped with a Falcon III direct electron detector camera. Electron micrographs revealed low contrast of 4F2hc-LAT2 particles, even at high defocus values. This is common in cryo-EM for relatively small proteins, rendering the use of a Volta phase plate (VPP) for contrast improvement beneficial [17]. Cryo-EM images were therefore recorded with the same electron microscope configuration and an activated VPP (Table S1). Dose-fractioned images were motion-corrected and the dataset was then refined by deselecting bad images, e.g., visually obviously ice-contaminated images and images with contrast transfer function (CTF) parameters outside the boundaries [21]. Remaining micrographs revealed a homogenous distribution of 4F2hc-LAT2 particles as exemplified by Figure 2A. These particles show different views, of which the length of the longest axes ranges from 100 to 120 Å (Figure 2A, black arrows). Some particles were substantially larger and elongated, and their longer axes ranged from 180 Å to 200 Å in length (Figure 2A, white arrows). These larger particles may be attributed to dimers of 4F2hc-LAT2 complexes, as seen using BN-PAGE (Figure 1). During the particle picking procedure, these larger particles were excluded and only 4F2hc-LAT2 heterodimeric particles were picked and used for subsequent rounds of reference-free 2D-classification. Representative examples of 2D-class averages are shown in Figure 2B depicting projections of the human 4F2hc-LAT2 heteromeric complex viewed from different angles. 4F2hc-LAT2 projections reflecting views from the membrane plane clearly demonstrate the presence of a smaller and a larger density (Figure 2B, 2D-class averages marked with a red asterisk) comparable to the previously reported 2D-class averages based on negatively stained 4F2hc-LAT2 particles [7,13]. An initial 3D-model was calculated applying the recently implemented stochastic-gradient-descent algorithm of the RELION software [22]. After 3D-classification and several rounds of 3D-refinement, the final cryo-EM 3D-map was calculated from 47,933 particles. The resolution was determined to 14.3 and 12.9 Å using the Fourier shell correlation (FSC) cut-off-criterion of 0.5 and 0.143 (gold standard), respectively (Figure 3A; Table S1) [23,24]. The obtained cryo-EM 3D-map resolution of ~13 Å using a 200 kV FEI Tecnai F20 electron microscope is moderate and should be significantly improved using a cutting-edge microscope such as a Titan Krios. Plotting the angular distribution of particles used for the final 3D-map calculation revealed a relatively even angular particle distribution (Figure 3B).

**Figure 4:**
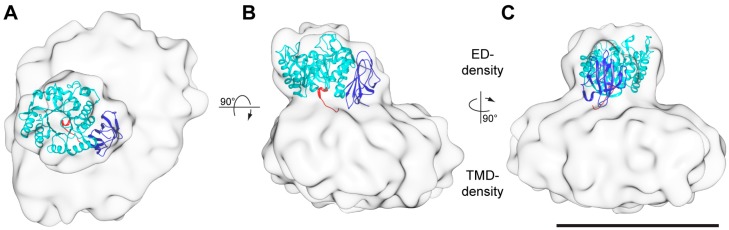
Cryo-EM 3D-map at a resolution of ~13 Å. The 3D-map is displayed as viewed from the extracellular side (**A**) and from the membrane plane (**B**,**C**). The 3D-map is composed of two densities. The smaller and the larger densities are named ED- and TMD-density, respectively. The structure of the 4F2hc-ED (PDB ID code 2DH2, [6]) was fitted into the ED-density and is shown as ribbon model. The 4F2hc-ED structure is composed of an N-terminal region (red), a TIM-barrel subdomain (cyan), and a C-terminal eight antiparallel β-strand subdomain (blue). The N-terminus (red) also reflects the approximate location of the disulfide bridge between 4F2hc and LAT2. All 3D-maps are equally scaled and the scale bar in panel C represents 100 Å.

Consistent with the previously obtained negative-stain EM 3D-maps of 4F2hc-LAT2 [7,13], the here presented cryo-EM 3D-map consists of two differently sized densities (Figure 4). Whereas the smaller density was identified as the 4F2hc-ED density (ED-density), the larger one was attributed to LAT2 plus the N-terminal cytoplasmic domain and the one TMD of 4F2hc, and co-purified lipids embedded in a LMNG/CHS micelle (TMD-density). If viewed from the extracellular side, the ED-density is laterally shifted by ~27 Å from the center of the TMD-density towards its edge (Figure 4A). Compared to the membrane region of 4F2hc-LAT2, the ED of 4F2hc was well-defined. Thus, the X-ray structure of 4F2hc-ED (PDB ID code 2DH2, [6]) could nicely be fitted into the ED-density of the cryo-EM 3D-map applying the volume-fit-procedure implemented in the Chimera software [25]. The best fit of the 4F2hc-ED structure with the ED-density was obtained if, (i), the N-terminal region (Figure 4, red colored ribbon) faces the TMD-density, (ii), the TIM-barrel subdomain (Figure 4, cyan colored ribbon) is located peripherally and, consequently, (iii), the eight antiparallel β-sheet containing C-terminal subdomain (Figure 4, blue colored ribbon) aligns with the center of the TMD-density. This architecture tilts the 4F2hc-ED structure by approximately 20° with respect to the membrane plane. 4F2hc is composed of a small cytoplasmic domain, one TMD, and a large ED. The N-terminal region of the 4F2hc-ED structure thus represents the location of the disulfide bridge between 4F2hc and LAT2. In the 3D-map, this region is laterally shifted with respect to the center of the TMD-density (Figure 4A,B, red colored ribbon). Consequently, this model corroborates the observation based on the homology model of human LAT2 (LAT2^HM^), that the Cys-residue involved in intersubunit disulfide bridge formation (Cys-154) is located in a peripheral loop between the predicted TMDs 3 and 4 of LAT2^HM^ [7].

The larger density of the cryo-EM 3D-map, the TMD-density, reflects LAT2, the small cytoplasmic domain, the one TMD of 4F2hc, and co-purified lipids embedded in a LMNG/CHS detergent micelle. The TMD-density is approximately 145 Å in length, 115 Å in width, and 60 Å in height, and differs in appearance to the published negatively-stained TMD-density [7], which is approximately 100 Å in length, 60 Å in width, and 60 Å in height. The difference in length and width but not in height between the TMD-densities is attributed to the use of different purification detergents for structure determination resulting in differently sized micelles [26]. Moreover, the here described 3D-map originates from cryo-EM rather than negative-stain EM [7,13], where negative staining with heavy metal salt and drying of the sample are applied causing micellar size reduction [27].

**Figure 5:**
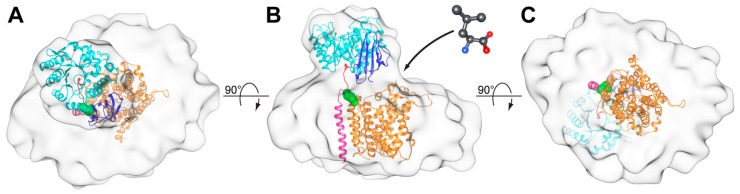
Structural model of the 4F2hc-LAT2 heterodimeric complex based on the cryo-EM 3D-map. The model is shown from the extracellular side (**A**), the membrane plane (**B**), and the cytoplasmic side (**C**). The structure of 4F2hc-LAT2 is displayed as ribbon representation, and the structural elements of 4F2hc-ED (PDB ID code 2DH2) are colored as in Figure 4 (N-terminal region, red; TIM-barrel subdomain, cyan; C-terminal eight antiparallel β-strand subdomain, blue). Whereas the modeled TMD of 4F2hc is shown in magenta, the LAT2 homology model (LAT2^HM^) is presented in orange. Individual atoms of the disulfide bridge between 4F2hc and LAT2 are shown as green spheres. In panel (**B**) the putative substrate entry side is represented by an L-Leu molecule (grey sticks) and an arrow.

In order to better understand the molecular architecture of 4F2hc-LAT2, we modeled the heterodimeric structure based on our cryo-EM 3D-map, the fitted 4F2hc-ED structure (PDB ID code 2DH2, [6]), and LAT2^HM^, which is based on the structure of a bacterial l-arginine/agmatine antiporter (amino acid sequence identity to LAT2: ~20%) [7]. The modeled 4F2hc-LAT2 structure revealed an elongated arrangement of TMDs, which is best viewed from the cytoplasmic side (Figure 5).

## 3. Materials and Methods

### 3.1. Cloning, Overexpression, and Purification of Human 4F2hc-LAT2

The generation of the *Pichia pastoris* human 4F2hc-LAT2 overexpression strain was reported previously [12]. For protein production, a preculture of this strain in yeast-extract-peptone-dextrose (YPD)-media containing Zeocin (1 µg/mL, Sigma, St. Louis, MO, USA) was used to inoculate buffered complex glycerol medium (BMGY), and the culture was grown at 30°. At OD_600_ ~50, the medium was changed to the buffered complex methanol expression medium (BMMY) containing 1% (*v*/*v*) of methanol. The culture was allowed to grow for another 28 h at 30 °C before harvesting using centrifugation at 10,000× *g*, 4 °C for 10 min. The cells were resuspended in 50 mM Na-phosphate pH 7.4, 10% (*v*/*v*) glycerol, 1 mM EDTA (ethylenediaminetetraacetic acid), and lysed using 5 microfluidizer (Microfluidics) cycles at 1500 bar. Cell debris were removed using centrifugation at 10,000× *g*, 4 °C for 10 min, and membranes were collected using ultracentrifugation at 150,000× g, 4°C for 1 h. The crude membranes were homogenized in 80 mM Bis-Tris propane pH 6.8, 300 mM NaCl, 10% (*v*/*v*) glycerol, collected again using another ultracentrifugation round, homogenized, diluted with 80 mM Bis-Tris propane pH 6.8, 300 mM NaCl, 10% (*v*/*v*) glycerol to a concentration of 300 mg membranes per mL, and finally flash frozen and stored at −75 °C until further use.

The 4F2hc-LAT2 heteromeric complex was purified as reported previously using Ni-NTA affinity chromatography [7,12,13]. The heteromeric complex was eluted from the Ni-NTA resin with 20 mM Bis-Tris propane pH 6.8, 150 mM NaCl, 2% (*v*/*v*) glycerol, 0.01% (*w*/*v*) LMNG/CHS (5/1; *w*/*w*), 10 mM oxidized gluthathione, and 200 mM l-histidine, yielding 40 µg of pure 4F2hc-LAT2 heterodimeric complex per g of wet *P. pastoris* cells.

### 3.2. BN-PAGE and Western Blot Analysis

For BN-PAGE and Western blot analysis, 4–16% gradient gels (Invitrogen, Carlsbad, CA, USA) were used and 5 µg of purified 4F2hc-LAT2 loaded per lane. Electrophoresis was carried out as described by the protocol of the gel manufacturer. For Western blot analysis, the protein was transferred to a polyvinylidene difluoride (PVDF) membrane (Immobilon-P, Sigma). The membrane was blocked with 50 mM Tris-HCl pH 8.0, 150 mM NaCl, and 0.05% (*v*/*v*) Tween-20 containing 3% (*w*/*v*) bovine serum albumin (BSA) (Sigma). For anti-His detection the Penta-His^TM^ Antibody (Qiagen, Hilden, Germany) was used as primary antibody and goat-anti-mouse IgG (H+L)-HPR conjugate (Bio-Rad, Hercules, CA, USA) as secondary antibody. For anti-Strep detection the StrepMAB-Classic HPR-conjugate (iba, Göttingen, Germany) was used. For the visualization of antibody labeled bands, the PVDF membranes were treated with ECL^TM^ solution according to the protocol of the manufacturer (GE Healthcare, Chicago, IL, USA).

### 3.3. Grid Preparation and Cryo-EM Data Collection

Subsequently, after purification, the protein was diluted to 300 µg/mL with 20 mM Bis-Tris propane pH 6.8, 150 mM NaCl, 2% (*v*/*v*) glycerol, 0.01% (*w*/*v*) LMNG/CHS (5/1; *w*/*w*), 10 mM oxidized gluthathione, 200 mM l-histidine, and cryo-EM grids (R1.2/1.3 grids containing a 2 nm carbon layer; Quantifoil) were prepared by adsorbing 2.5 µL of protein solution for 3 s on the carbon layer of the glow discharged grids (12 s, 10 mA, 0.25 mbar) followed by three consecutive grid washing steps with 200 µL ultrapure H_2_O for 10 s each. The grid was then plunge frozen in liquid ethane after blotting off the excess water for 1 s using a Vitrobot Mark IV apparatus operated at approximately 100% humidity and cooled to 4–5 °C. The grids were stored in liquid nitrogen until further use.

Cryo-EM data was collected using a 200 kV FEI Tecnai F20 electron microscope equipped with a Falcon III direct electron detector camera and VPP at a magnification of 100,000× and at a defocus range of −0.6 to −1.2 µm. Data was collected in an automated fashion using the EPU software (FEI). Images were recorded for 3 s with a frame exposure time of 77 ms and a dose of 1.9 e^−^/A^2^/frame, resulting in a total accumulated dose on the specimen level of approximately 74 e^−^/Å^2^ per exposure.

### 3.4. Calculation of the Cryo-EM 3D-Map

Dose-fractioned images were motion-corrected and frames dose-weighted using MotionCor2 [28]. The contrast transfer function (CTF) parameters were estimated using ctffind 4.1.10 [29]. Images of bad quality, e.g., strong drift, resolution above 6 Å, or phase shifts below 45° and above 135° according to CTF [21], and ice-contaminated images were selected and discarded using FOCUS software [30]. The remaining 691 images were used for image processing with RELION 3 [22]. An initial set of 3,297 particles from a small set of images were picked by the Laplacian-of-Gaussian filter using the auto-picking procedure of RELION 3. 2D-class averages were calculated, and those selected were used to auto-pick 235,830 particles. Particles belonging to low-abundance classes were removed by 4 rounds of 2D-classification. The remaining particles were used to generate an initial model calculated using the stochastic-gradient-descent algorithm of RELION 3 and used for a first round of 3D-classification. An iterative procedure was employed for subsequent 3D-classification and refinement rounds using the best output 3D-maps. Finally, a single 3D-class, including 47,933 particles, was subjected to auto-refinement in RELION. The FSC of the final unmasked 3D-map was calculated by applying the postprocess procedure implemented in the RELION software. Based on the FSC curve, the resolution was determined at the 0.5 and 0.143 (gold standard) FSC-cut-off-criteria to 14.3 Å and 12.9 Å, respectively.

### 3.5. Modeling the 4F2hc-LAT2 Structure

The rotational and translational position of the LAT2 homology model (LAT2^HM^) [7] in a membrane was calculated using the positioning of proteins in membrane (PPM) server [31]. The procedure for positioning of the LAT2^HM^ into the cryo-EM TMD-density was done as follows: First, the center of mass of the LAT2^HM^ was placed in the center of the TMD-density. Second, based on the PPM-server output, the respective vertical axes of the LAT2^HM^ and the TMD-density were aligned. Third, the LAT2^HM^ was rotated along the vertical axes such that the Cys residues of 4F2hc and LAT2 involved in disulfide bridge formation horizontally align. In order to visualize the TMD of 4F2hc, the fitted 4F2hc-ED structure was N-terminally extended by modeling a straight, with respect to the membrane plane orthogonal 29 Ala residues containing and ~42 Å long α-helix using COOT [32].

### 3.6. Figure Preparation

Molecular graphics and analyses were performed with UCSF Chimera [25].

## 4. Conclusions

In this study we purified the in *Pichia pastoris* heterologously overexpressed human 4F2hc-LAT2 heterodimeric complex. The protein complex was obtained pure and correctly assembled based on BN-PAGE and cryo-EM. Furthermore, we have determined the first 3D cryo-EM structure of the human 4F2hc-LAT2 heterodimeric complex at ~13 Å resolution. The usage of the VPP for cryo-EM data collection was deemed crucial due to otherwise too low image contrast. The structure revealed two major densities of which the smaller one was identified as the 4F2hc-ED. The larger, micellar density is therefore composed of LAT2 plus the cytoplasmic domain and the TMD of 4F2hc and co-purified lipids embedded in a LMNG/CHS micelle. The X-ray structure of 4F2hc-ED nicely fitted into the smaller density (Figure 4; ED-density and Figure 5). This experimentally revealed that 4F2hc-ED is laterally shifted by ~27 Å from the center of the micellar density and tilted by ~20° with respect to LAT2 and the membrane plane. The larger density, the TMD-density, resembled an oblate ellipsoid. In order to investigate the TMD-density further, we generated an in silico model of the 4F2hc-LAT2 structure based on our cryo-EM 3D-map. In this model, the LAT2^HM^ is located in the center of the TMD-density and positioned such that the Cys residues of 4F2hc-ED and the LAT2^HM^ responsible for intersubunit disulfide bridge formation are in close proximity (Figure 5, green spheres). In addition, the one TMD of 4F2hc was modeled (Figure 5, magenta colored α-helix). With this, an elongated arrangement of all 13 TMDs of 4F2hc-LAT2 is discerned (Figure 5C). As a consequence, this TMD arrangement may explain why the TMD-density resembles an oblate ellipsoid. In addition, the presented architecture of the 4F2hc-LAT2 structure provides a substrate entry point and would therefore allow for relatively unhindered substrate binding to the catalytic HAT subunit LAT2 (Figure 5B). In summary, the here presented cryo-EM 3D-map of human 4F2hc-LAT2 provided new insights on the molecular architecture of this complex revealing the relative position of 4F2hc with respect to LAT2 and the membrane plane under near native conditions. Moreover, this work paves the way for high-resolution structure solution using cryo-EM of human 4F2hc-LAT2 and HATs in general.

## Supplementary Materials

Supplementary Table 1 (Table S1) can be found at https://www.mdpi.com/1422-0067/20/4/931/s1.

## Abbreviations

3D	three-dimensional
BN-PAGE	blue native-polyacrylamide gel electrophoresis
CHS	cholesteryl hemisuccinate
CTF	contrast transfer function
ED	ectodomain
EM	electron microscopy
FSC	Fourier shell correlation
HAT	heteromeric amino acid transporter
ID	identifier
LAT	L-type amino acid transporter
LAT2^HM^	homology model of human LAT2
LMNG	lauryl maltose neopentyl glycol
MW	molecular weight
PDB	protein databank
SLC	solute carrier
TIM	triosephosphate isomerase
TMD	transmembrane domain
VPP	Volta phase plate
