# Supplementary material for: Volta Phase Plate Cryo-EM Structure of the Human Heterodimeric Amino Acid Transporter 4F2hc-LAT2
Source: Int J Mol Sci. 2019 Feb 21;20(4):931. doi: 10.3390/ijms20040931 (PMC6413005; doi:10.3390/ijms20040931)
Supplement: Supplementary file 1 [file ijms-20-00931-s001.pdf]

Table S1. Data collection and processing

Electron microscope	200 kV FEI Tecnai F20
Detector	Falcon III
Volta phase plate used	Yes
Magnification	100,000x
Pixel size (Å/pixel)	1.052
Electron dose (e^- / $Å^2$ / frame)	1.9
Frames (No.)	39
Total electron dose per movie (e^- / $Å^2$)	74
Defocus range (µm)	-0.6 to -1.2
Symmetry imposed	C1
Initial particle images (No.)	235,830
Final particle images (No.)	47,933
3D Map resolution (FSC threshold)	14.3 Å (0.5); 12.9 Å (0.143, Gold standard)
